# *SLC26A9* Gene Is Associated With Lung Function Response to Ivacaftor in Patients With Cystic Fibrosis

**DOI:** 10.3389/fphar.2018.00828

**Published:** 2018-07-26

**Authors:** Harriet Corvol, Julie Mésinèle, Isman-Hassan Douksieh, Lisa J. Strug, Pierre-Yves Boëlle, Loïc Guillot

**Affiliations:** ^1^Centre de Recherche Saint-Antoine (CRSA), Sorbonne Université, UPMC Univ Paris 06, INSERM, Paris, France; ^2^Pneumologie Pédiatrique, APHP, Hôpital Trousseau, Paris, France; ^3^INSERM, UMR_S 1136, Institut Pierre Louis d’Epidémiologie et de Santé Publique, Sorbonne Université, UPMC Univ Paris 06, Paris, France; ^4^Program in Genetics and Genome Biology, Research Institute, The Hospital for Sick Children, Toronto, ON, Canada

**Keywords:** cystic fibrosis, lung, gene modifier, SLC26A9, ivacaftor, pharmacogenetic, individualized medicine

## Abstract

Ivacaftor is a drug used to treat cystic fibrosis (CF) patients carrying specific gating *CFTR* mutations. Interpatient variability in the lung response has been shown to be partly explained by rs7512462 in the Solute Carrier Family 26 Member 9 (*SLC26A9*) gene. In an independent and larger cohort, we aimed to evaluate whether *SLC26A9* variants contribute to the variability of the lung phenotype and if they influence the lung response to ivacaftor. We genotyped the French CF Gene Modifier Study cohort (*n* = 4,840) to investigate whether *SLC26A9* variants were involved in the lung phenotype heterogeneity. Their influence in the response to ivacaftor was tested in the 30 treated patients who met the inclusion criteria: older than 6 years of age, percent-predicted forced expiratory volume measured in 1 s (FEV_1pp_) in the 3 months before treatment initiation ranging between 40 and 90%. Response to treatment was determined by the change in FEV_1pp_ from baseline, averaged in 15–75 days, and the 1st-year post-treatment. We observed that *SLC26A9* variants were not associated with lung function variability in untreated patients and that gain of lung function in patients treated with ivacaftor was similar to clinical trials. We confirmed that rs7512462 was associated with variability in ivacaftor-lung response, with a significant reduction in lung function improvement for patients with the C allele. Other *SLC26A9* SNPs also contributed to the ivacaftor-response. Interindividual variability in lung response to ivacaftor is associated with *SLC26A9* variants in French CF patients. Pharmacogenomics and personalized medicine will soon be part of CF patient care.

## Introduction

Cystic fibrosis (CF) is the most common, severe, autosomal recessive genetic disease in Caucasians. It is caused by mutations in the gene encoding the CF transmembrane conductance regulator (CFTR), a chloride channel expressed in epithelial cells throughout the body (Riordan et al., 1989). The disease affects several organs such as the lungs, pancreas, intestine, and liver. Over 2,000 variations in the *CFTR* gene have been described, including 312 CF-causing variant [The Clinical and Functional TRanslation of CFTR (CFTR2)^1^], which are usually classified into six classes, according to their resulting effect on the protein (Corvol et al., 2016). The most common mutation (70% of alleles) is p.Phe508del (F508del), which prevents normal CFTR expression at the apical surface of epithelia. *CFTR* genotype strongly influences pancreatic function, which is either deficient (PI for pancreatic insufficiency), or normal (PS for pancreatic sufficiency). It is recognized that in the major part of cases, patients carrying two PI-associated severe *CFTR* mutations have a classical form of CF, whereas others have a milder form of disease associated with PS (Corvol et al., 2016).

Until recently, treatment of CF was only symptomatic. However, in recent years, considerable efforts have led to the development of therapies that target the CFTR protein. Since 2012, patients carrying the *CFTR* gating mutation p.Gly551Asp (G551D) and who are older than 6 years can be treated with ivacaftor, a molecule called a potentiator, which targets CFTR directly to increase the probability of the channel being open (Van Goor et al., 2009). Significant clinical benefits of ivacaftor, such as gain of lung function and reduced number of pulmonary exacerbations, were initially observed in patients older than 12 years and carrying at least one G551D *CFTR* mutation (Ramsey et al., 2011). Subsequently, ivacaftor was approved for other *CFTR*-gating mutations: p.Gly1244Glu (G1244E), p.Gly1349Asp (G1349D), p.Gly178Arg (G178R), p.Gly551Ser (G551S), p.Ser1251Asn (S1251N), p.Ser1255Pro (S1255P), p.Ser549Asn (S549N), and p.Ser549Arg (S549R) (De Boeck et al., 2014) and younger patients (Davies et al., 2013). Now, ivacaftor is approved for patients with CF older than 2 years carrying at least one of these gating mutations (Davies et al., 2016).

The Solute Carrier Family 26 Member 9 gene, *SLC26A9*, was recently shown to modulate the airway response to CFTR-directed therapeutics. In particular, in CF patients carrying at least one CFTR-G551D mutation, the single nucleotide polymorphism (SNP), rs7512462, in the *SLC26A9* gene was shown to explain 28% of the response variability to ivacaftor (Strug et al., 2016). In that study, rs7512462 was also associated with the lung function variability of patients carrying a *CFTR*-gating mutation. Moreover, *SLC26A9* variants have been previously shown in genome wide association studies (GWAS) to contribute to the phenotype variability of meconium ileus (Sun et al., 2012) (rs7512462, rs4077468, rs4077469, rs7419153, rs12047830, rs12741299) and CF-related diabetes (CFRD, rs4077468, rs4077469, rs1874361) (Blackman et al., 2013).

In the current study, we examine the French cohort (*n* = 4,840) of the French CF Gene Modifier Study to investigate whether *SLC26A9* variants firstly contribute to the variability of the lung phenotype, and secondly influence the response to ivacaftor.

## Materials and Methods

### Study Subjects and Lung Phenotype

Patients with CF treated in 38 out of the 47 French CF centres between January 2004 and January 2017 were enrolled in the French CF Modifier Gene Study. As of January 1, 2017, 4,840 patients with CF had been included (corresponding to ∼75% of all French patients with CF) (Vaincre la Mucoviscidose and Ined, 2017). The study was approved by the French ethical committee (CPP n°2004/15), and the information collection was approved by the Commission Nationale de L’informatique et des Libertés (n°04.404). Informed consent in writing was obtained from each patient and/or guardian.

Measurements of the forced expiratory volume measured in 1 s (FEV_1_) were either expressed as percent-predicted values (FEV_1pp_) using the Global Lung Function Initiative (GLI) equations (Quanjer et al., 2012) or transformed to the Kulich Normalized Mortality Adjusted CF-specific lung phenotype (SaKnorm *Z*-value) (Kulich et al., 2005; Taylor et al., 2011). This quantitative phenotype allows indeed direct comparison of lung phenotypes between patients with CF and accounts for differential survival.

**FIGURE 1:**
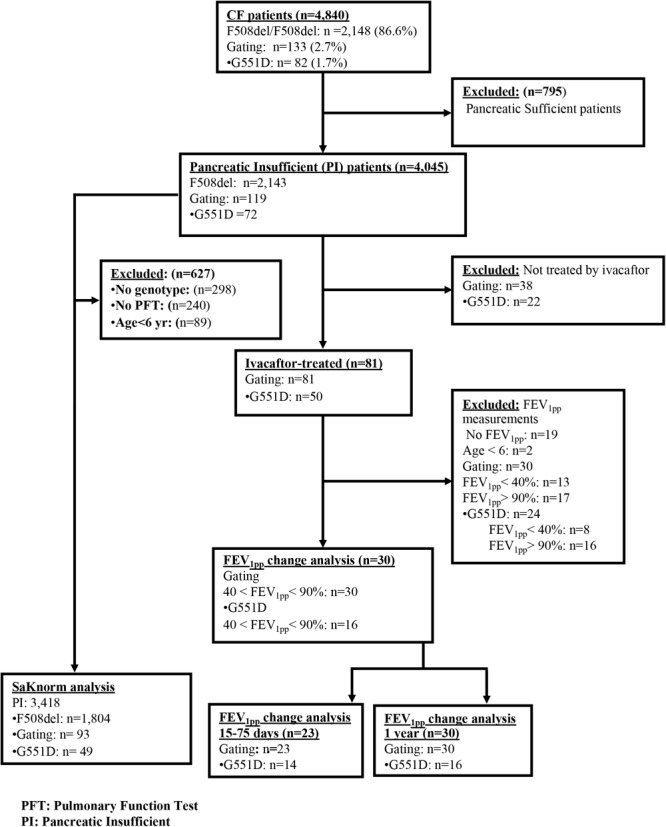
Flowchart of the study.

Details on the 4,840 CF patients are reported in **Table 1** and in the Flowchart (**Figure 1**). Only patients with severe *CFTR* mutations were considered (pancreatic sufficient patients excluded). Among those, 119 carried at least one gating mutation for which ivacaftor therapy has been approved in Europe (i.e., G551D, G1244E, G1349D, G178R, G551S, S1251N, S1255P, S549N, and S549R), 81 were prescribed ivacaftor. Finally, 60 patients on ivacaftor had lung function measurements available before and after treatment initiation. To assess the association of *SLC26A9* with lung function response to ivacaftor, we included the 30 patients older than 6 years of age and with FEV_1pp_ in the 3 months before treatment initiation ranging between 40 and 90%; their *CFTR* genotypes are depicted in Supplementary Table S1. The response to treatment was determined by the change in FEV_1pp_ from baseline, averaged in the 15–75 days after treatment, as well as that averaged over the 1st-year post-treatment, as used in an earlier study (**Table 1**) (Strug et al., 2016). Besides, these two timelines were chosen to evaluate: (1) an “early” response (15–75 days), as it takes several days for ivacaftor to reach a maximal response; and (2) a “long-term” response (1 year), computed by averaging the FEV_1pp_ over the 1st year of ivacaftor treatment, illustrating the overall response of the patients.

**Table 1:** Baseline characteristics of the patients.

Patient characteristics	*CFTR* variants		
	F508del/ F508del	Gating^$^/ other	G551D/ other
Pancreatic Insufficient (*n* = 4,045)	*n* = 2,143	*n* = 119	*n* = 72
Sex (male/female)	1,125/1018	64/55	42/30
European origin, *n* (%)	2,061 (96)	105 (88)	67 (93)
Treated by ivacaftor (*n* = 81)			
FEV_1pp_ (baseline) ^$^		*n* = 60	*n* = 40
>90%		17	16
40–90%		30	16
<40%		13	8
FEV_1pp_ change analysis (*n* = 30)		*n* = 30	*n* = 16
Age, mean (*SD*)		20.0 (14.0)	17.6 (12.4)
Sex (male/female)		18/12	10/6

*^$^Ivacaftor-approved CFTR gating mutations: G551D, G1244E, G1349D, G178R, G551S, S1251N, S1255P, S549N, and S549R; ^∗∗^FEV_1pp_ (forced expiratory volume in 1 s percent-predicted) value in the 3 months before treatment. Data are means (SD) or numbers (%) unless otherwise indicated. CFTR: Cystic Fibrosis Transmembrane Conductance Regulator.*

### Genotyping

The genotyping of *SLC26A9* SNPs (rs7512462, rs4077468, rs7419153, rs12047830, rs4077469, rs12741299, and rs1874361) was carried out using Kompetitive Allele Specific PCR (KASP) genotyping chemistry (LGC, Teddington, United Kingdom).

### Statistical Analysis

Lung function was analyzed as FEV_1pp_ (GLI) (Quanjer et al., 2012) or CF-specific quantile-Z value (SaKnorm *Z*-value) (Kulich et al., 2005; Taylor et al., 2011). For each patient, FEV_1pp_ measurements in the 15-75 days following treatment initiation were averaged to determine the early response. We used the trapezoidal rule to compute average FEV_1pp_ over the 1st year to account for irregular measurements. The change in FEV_1pp_ from baseline was then analyzed by linear regression, adjusting for baseline measurement. We used additive coding to estimate the effect of SNPs in *SLC26A9.* Reference alleles were taken from annotations of the human genome^2^. Among SNPs in *SLC26A9*, rs7512462 had previously shown association with treatment response and, therefore, was analyzed independently from the other SNPs. We also analyzed five other SNPs in *SLC26A9*, adjusting the *P*-values for multiplicity in this situation.

Conformance of the allele frequencies with the Hardy–Weinberg equilibrium (HWE) was tested using a Fisher’s exact test. As shown in Supplementary Table S2, the population did not deviate significantly from the HWE indicating no issue with the genotyping method or population stratification.

We reconstructed haplotypes using the EM algorithm with all patients (*n* = 4,045) keeping loci in their physical order on chromosome 1 (using haplo.stats package in the R software) (Supplementary Table S3) (Lake et al., 2003). We analyzed the association of haplotypes with FEV_1pp_ using additive haplotype coding (see Supplementary Material).

A *P*-value of less than 5% was interpreted as evidence of a statistically significant difference or association. Multiple comparisons utilized the Bonferroni correction. All association analyses were carried out using the software, R (version 3.4.0^3^).

## Results

### *SLC26A9* Gene Variants and Lung Function

**FIGURE 2:**
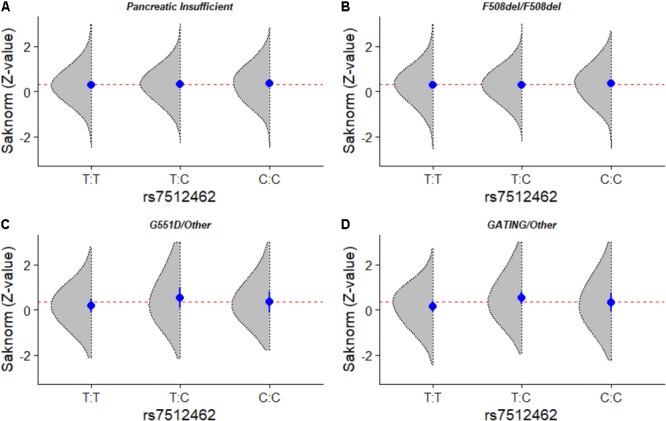
Lung phenotype distribution according to *SLC26A9* rs7512462 variant in patients with cystic fibrosis. Smoothed histograms of SaKnorm *Z*-values by *SLC26A9* rs7512462 genotypes (gray areas), genotype specific mean ± SE (dot and bars) and overall mean (dashed). **(A)** All pancreatic insufficient CF patients (*n* = 3,418), **(B)** *CFTR* F508del homozygous CF patients (*n* = 1,804), **(C)** CF patients with at least one G551D allele (*n* = 49) and **(D)** CF patients with at least one ivacaftor-approved gating mutation (*n* = 93).

In the absence of ivacaftor treatment, the effect of rs7512462 on lung function did not reach statistical significance for any *CFTR* genotype group. We considered all patients (*n* = 3,418), F508del homozygous patients (*n* = 1,804), carriers of at least one ivacaftor-approved gating mutation (*n* = 93), and carriers of one G551D allele (*n* = 49) (**Figure 2** and **Table 2**). The results were similar with other SNPs in the *SLC26A9* gene (i.e., rs1874361, rs12741299, rs4077468, rs4077469, rs12047830, rs7419153) (**Table 3**).

**Table 2:** Mean differences in lung function according to *CFTR* and *SLC26A9* variants.

	All pancreatic insufficient		F508del/F508del		G551D/other		Gating^$^/other	
	patients (*n* = 3,418)		(*n* = 1,804)		(*n* = 49)		(*n* = 93)	
*SLC26A9*	Mean change	*P*-value	Mean change	*P*-value	Mean change	*P*-value	Mean change	*P*-value
variants^$^	in CF SaKnorm^∗^		in CF SaKnorm^∗^		in CF SaKnorm^∗^		in CF SaKnorm^∗^	
rs7512462	0.03 ± 0.02	0.20	0.03 ± 0.03	0.29	0.14 ± 0.16	0.40	0.13 ± 0.12	0.27
rs1874361	–0.02 ± 0.02	0.30	–0.00 ± 0.03	0.97	–0.07 ± 0.16	0.66	–0.17 ± 0.11	0.13
rs12741299	0.01 ± 0.03	0.70	–0.02 ± 0.04	0.64	–0.06 ± 0.33	0.86	0.23 ± 0.23	0.32
rs4077468	0.05 ± 0.02	0.01	0.05 ± 0.03	0.07	0.22 ± 0.18	0.22	0.14 ± 0.13	0.29
rs4077469	0.05 ± 0.02	0.01	0.05 ± 0.03	0.08	0.22 ± 0.17	0.21	0.14 ± 0.13	0.26
rs12047830	0.04 ± 0.02	0.03	0.03 ± 0.02	0.19	0.37 ± 0.18	0.05	0.27 ± 0.13	0.05
rs7419153	–0.02 ± 0.02	0.359	–0.02 ± 0.03	0.47	–0.35 ± 0.18	0.06	–0.18 ± 0.14	0.20

*^$^See description Supplementary Table S2; ^$^Ivacaftor-approved CFTR gating mutations: G551D, G1244E, G1349D, G178R, G551S, S1251N, S1255P, S549N, and S549R; ^∗^change per minor allele (linear regression of SaKnorm Z-value with additive model). SaKnorm is normalized function of FEV_1_ (forced expiratory volume in 1 s) adjusted for age, sex, height, and cohort-specific survival.*

**Table 3:** Change in lung function evolution after 15–75 days and after 1 year on ivacaftor.

	*n*	Before^∗∗^	After	Difference	95% CI	*P*-value^∗^
**Average FEV_1pp_ measures within 15–75 days on ivacaftor**						
Gating^$^/other	23	66.13	80.85	11.72	7.32–16.06	<0.0001
G551D/other	14	70.66	85.05	14.39	7.89–20.66	0.00012
**Average FEV_1pp_ measures in the 1st year on ivacaftor**						
Gating^$^/other	30	69.07	78.90	9.83	4.91–14.77	<0.0001
G551D/other	16	69.14	84.2	15.06	7.10–23.14	<0.0001

*^$^Ivacaftor-approved CFTR gating mutations: G551D, G1244E, G1349D, G178R, G551S, S1251N, S1255P, S549N, and S549R; ^∗∗^FEV_1pp_ (forced expiratory volume in 1 s percent-predicted) value in the 3 months before treatment. ^∗^Paired Mann–Whitney–Wilcoxon test.*

### Change in FEV_1_ With Ivacaftor

**FIGURE 3:**
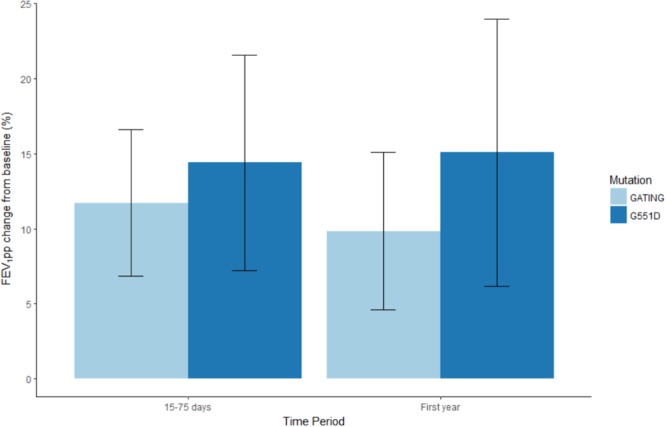
FEV_1pp_ change from baseline in patients carrying at least one ivacaftor-approved gating mutation (light blue) or one G551D variant (dark blue) within the first 15–75 days and over the 1st year of ivacaftor.

In total, 30 pancreatic insufficient CF patients had at least one ivacaftor-approved gating mutation, baseline FEV_1pp_ between 40 and 90%, and post-treatment data (see Flowchart, **Figure 1**). FEV_1pp_ measurements within 15–75 days post-treatment and in the 1st year on ivacaftor were available for 23 and 30 patients, respectively, among whom 14 and 16, respectively, were carriers of at least one G551D allele (see Flowchart, **Figure 1**). Overall, patients showed an improvement in FEV_1pp_ after 15–75 days and in the 1st year on ivacaftor. For individuals with ivacaftor-approved gating mutations, the baseline-adjusted change in FEV_1pp_ was +11.72% (95% CI: 7.32–16.06) at 15–75 days and +9.83% (95% CI: 4.91–14.77) in the 1st year of treatment (*P* < 0.0001, **Table 3** and **Figure 3**). In patients with at least one G551D allele, the improvement in FEV_1pp_ was +14.39% (95% CI: 7.89–20.66) at 15–75 days and +15.06% (95% CI: 7.1–23.14) over the 1st year of treatment (*P* < 0.0001, **Table 3** and **Figure 3**).

### *SLC26A9* Variants and Ivacaftor Treatment Response

**FIGURE 4:**
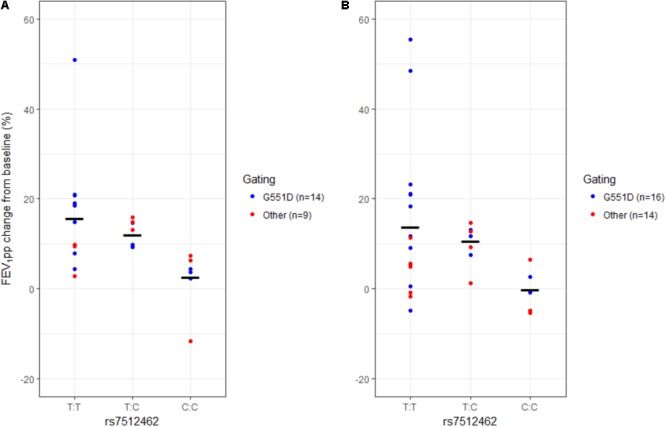
FEV_1pp_ change from baseline in patients with ivacaftor treatment according to rs7512462 *SLC26A9* genotype. Individual change in patients with at least one G551D mutation (blue) and with other gating mutations (red) and model fitted mean change (horizontal bar) within the first 15–75 days of treatment **(A)** and over the 1st year of treatment **(B)**.

In patients carrying at least one G551D allele, the response to treatment changed with the *SLC26A9* rs7512462 genotype, with less change in FEV_1pp_ (-7.7% over 15–75 days and -7.8% over 1 year of treatment) for each C allele (*P* = 0.0007 and 0.006, respectively; **Table 4** and **Figure 4**). Other *SLC26A9* variants showed similar associations with the changes in FEV_1pp_. In particular, the following SNPs demonstrated significant association after adjustment for multiplicity: with reduced FEV_1pp_ over 15–75 days: rs4077468, -7.9 FEV_1pp_ (*P*_adj_ = 0.0007); and rs4077469, -9.3 FEV_1pp_ (*P*_adj_ = 0.0035); or with increased FEV_1pp_ over 15–75 days: rs7419153, +9.8 FEV_1pp_ (*P*_adj_ = 0.0049) (**Table 4**).

**Table 4:** Change in FEV_1pp_ within 15–75 days and over 1 year on ivacaftor treatment according to *SLC26A9* variants in patients carrying at least one G551D mutation.

Period of evaluation	15–75 days after ivacaftor treatment start			Over the 1st year of ivacaftor treatment		
*SLC26A9* variants^$^	G551D/other (*n* = 14)			G551D/other (n = 16)		
SNP	FEV_1pp_ change^∗^	*P*-value	Adjusted *P*-value^∗∗^	FEV_1pp_ change^∗^	*P*-value	Adjusted *P*-value^∗∗^
rs7512462	–7.7 ± 1.7	0.0007	0.0049	–7.8 ± 2.4	0.0063	0.0441
rs1874361	4.4 ± 1.5	0.0107	0.0749	3.1 ± 3.0	0.3129	1.0000
rs12741299	2.9 ± 6.0	0.6336	1.0000	2.1 ± 5.9	0.7331	1.0000
rs4077468	–7.9 ± 1.3	0.0001	0.0007	–8.4 ± 1.7	0.0003	0.0027
rs4077469	–9.3 ± 1.9	0.0005	0.0035	–10.5 ± 2.3	0.0006	0.0042
rs12047830	–10.9 ± 2.7	0.0020	0.014	–7.5 ± 3.5	0.0518	0.3626
rs7419153	9.8 ± 2.1	0.0007	0.0049	8.1 ± 2.6	0.0089	0.0623

*^$^See description Supplementary Table S2; ^∗^linear regression of FEV_*1pp*_ (forced expiratory volume in 1 s percent-predicted) change with additive model adjusted on baseline; ^∗∗^Bonferroni adjustment.*

The results for individuals with gating mutations were similar. Indeed, the response to treatment also changed with the *SLC26A9* rs7512462 genotype, with less change in FEV_1pp_ (-5.9% over 15–75 days and -5.2% over 1 year of treatment) for each C allele (*P* = 0.0031 and 0.0042, respectively; **Table 5** and **Figure 4**). Moreover, other *SLC26A9* variants also showed similar associations with, in particular, significant association after adjustment for multiplicity for the following SNPs with reduced FEV_1pp_ over 15–75 days: rs4077468, -7.0 FEV_1pp_ (*P*_adj_ = 0.0231); and rs4077469, -7.8 FEV_1pp_ (*P*_adj_ = 0.0490) (**Table 5**).

**Table 5:** Change in FEV_1pp_ within 15–75 days and over 1 year on ivacaftor treatment according to *SLC26A9* variants in patients carrying at least one ivacaftor-approved *CFTR* gating mutation.

Period of evaluation	15–75 days after ivacaftor treatment start			Over the 1st year of ivacaftor treatment		
*SLC26A9* variants^$^	Gating^$^/other (*n* = 23)			Gating^$^/other (*n* = 30)		
SNP	FEV_1pp_ change^∗^	*P*-value	Adjusted *P*-value^∗∗^	FEV_1pp_ change^∗^	*P*-value^∗^	Adjusted *P*-value^∗∗^
rs7512462	–5.9 ± 1.7	0.0031	0.0217	–5.2 ± 1.8	0.0042	0.0294
rs1874361	3.7 ± 1.3	0.0120	0.0840	3.9 ± 1.9	0.0479	0.3346
rs12741299	–2.8 ± 4.8	0.5656	1.0000	–2.7 ± 4.2	0.5270	1.0000
rs4077468	–7.0 ± 2.1	0.0033	0.0231	–7.3 ± 2.2	0.0030	0.0210
rs4077469	–7.8 ± 2.6	0.0070	0.0490	–8.2 ± 2.6	0.0040	0.0280
rs12047830	–6.3 ± 2.4	0.0138	0.0966	–6.6 ± 2.6	0.0161	0.1127
rs7419153	6.3 ± 2.6	0.0233	0.1631	7.9 ± 2.5	0.0038	0.0266

*^$^See description Supplementary Table S2; ^∗^linear regression of FEV_1pp_ (forced expiratory volume in one second percent-predicted) change with additive model adjusted on baseline; ^∗∗^Bonferroni adjustment; ^$^Ivacaftor-approved CFTR gating mutations: G551D, G1244E, G1349D, G178R, G551S, S1251N, S1255P, S549N, and S549R.*

In these patients, the most frequent haplotypes (7 SNPs in physical order, Supplementary Table S2) were CCCGTAG (35%) and TACACGA (18%) (Supplementary Table S3). In agreement with the direction of association in the SNP analysis, FEV_1pp_ in carriers of at least one CCCGTAG haplotype increased on average by 17 ± 12% over 15–75 days and by 17 ± 17% over 1 year of treatment, while the FEV_1pp_ of the carriers of at least one TACACGA increased by 6 ± 5% over 15–75 days and 4 ± 5% over 1 year of treatment. Overall, this analysis did not provide significant evidence for a heterogeneity in FEV_1pp_ change with haplotypes (*P* = 0.2 over 15–75 days, *P* = 0.08 over 1 year), but was underpowered given the large diversity of haplotypes in the sample (Supplementary Table S3).

## Discussion

We have shown that the response to ivacaftor measured as lung function modulation varied between individuals and is associated with *SLC26A9* variants, as previously described (Strug et al., 2016). *SLC26A9* is a key candidate in CF as it has been shown to play a pleiotropic role across CF phenotypes, associated with meconium ileus (Sun et al., 2012; Li et al., 2014), immunoreactive trypsinogen at birth (Soave et al., 2014; Miller et al., 2015), pancreatic damage (Li et al., 2014), and CFRD (Blackman et al., 2013; Soave et al., 2014). With the development of new curative treatments, such as CFTR-targeted therapies, pharmacogenomics will become a major step toward functional personalized medicine (Corvol et al., 2016).

### *SLC26A9* Gene Modulates Ivacaftor Lung Response

We observed that *SLC26A9* variants were associated with the variability in lung responses to ivacaftor measured as FEV_1pp_ change over 1 year with treatment. Indeed, we have shown that, although the response varied between individuals, FEV_1pp_ improved after 15–75 days to 1 year on ivacaftor, in agreement with several clinical trials that led to the approbation of this drug (Ramsey et al., 2011; Davies et al., 2013, 2016; De Boeck et al., 2014; McKone et al., 2014). Further, we observed, as previously shown by Strug et al. (2016), that *SLC26A9* variants modulate this drug response. Focusing on *SLC26A9* rs7512462 in the patients carrying at least one G551D *CFTR* mutation, we observed that the CC genotype was associated with a decrease in FEV_1pp_ of -7.7%. Surprisingly, this effect was inverse to that observed in the pilot study of Strug et al. (2016) who showed an increase in FEV_1pp_ of approximately 8.5% for each additional C allele at rs7512462. Since rs7512462 is not presumed to be functional (Strug et al., 2016), it must be linked to the causal variants so that population-specific differences could explain this difference in direction. In fact, the North-American CF Gene Modifier consortium found significant population admixture in the North-American CF patients, with a large portion of patients reporting African–Caucasian, Mexican–Caucasian, and Indian–Caucasian ancestries (Li et al., 2011), whereby French patients are predominantly of Caucasian origin. Besides, it has been found that the pharmacogenetic response to drugs varied across ethnic groups, which might play a role in the differences observed here between French and Canadian cohorts (Corvol and Burchard, 2008). Nevertheless, Strug et al. (2016) observed in CF patients carrying at least one CFTR-G551D mutation, that the rs7512462 SNP in the *SLC26A9* gene explained 28% of the response variability to ivacaftor, a result similar to ours (22%) (Strug et al., 2016). We observed similar results when evaluating both G551D carriers and patients carrying other ivacaftor-approved gating mutations. There remains, however, a large part of interindividual variation besides the SNP status.

### *SLC26A9* Gene Is Not a Modifier of Lung Function in CF Patients

In this study, *SLC26A9* variants were not associated with variation in lung function of French patients with CF, regardless of their *CFTR* genotype (i.e., two copies of the F508del mutation and/or at least one gating mutation, the most frequent being G551D). These results are in agreement with previous, large international GWAS studies (Wright et al., 2011; Corvol et al., 2015). The latest and largest study, a meta-analysis of 6,365 French and North-American CF patients, identified five regions outside the *SLC26A9* locus that displayed significant association with variation in lung disease: the locus of the mucin genes *MUC4* and *MUC20*, of the solute carrier genes *SLC9A3* and *SLC6A14*, and of the HLA Class II region (Corvol et al., 2015). No association was observed between lung function and the *SLC26A9* gene. In the pilot study of Strug et al. (2016) *SLC26A9* rs7512462 also was not associated with lung function variation in CF patients who were homozygous for the F508del mutation. However, an association was observed for patients carrying at least one G551D variant, for whom the number of C alleles was positively associated with improved lung function (Strug et al., 2016). Another independent study of a smaller Brazilian CF cohort with various *CFTR* mutations also did not show an association of this variant with FEV_1_ heterogeneity (Pereira et al., 2017). We tested other *SLC26A9* variants that had been previously shown in GWAS to contribute to the variability of other phenotypes, such as meconium ileus (rs4077468, rs4077469, rs7419153, rs12047830, rs12741299) (Sun et al., 2012) and CFRD (rs4077468, rs4077469, rs1874361) (Blackman et al., 2013). However, we found that none of these variants contributed to the lung function heterogeneity in our CF cohort.

### Mechanisms of SLC26A9 Variants to Modulate Ivacaftor Lung Response

SLC26A9 is a highly conserved protein predominantly expressed in the lung (Lohi et al., 2002). It functions as a chloride channel with minimal bicarbonate conductance (Loriol et al., 2008; Bertrand et al., 2009), and may constitute an attractive alternative ion channel strategy to compensate for the CFTR defect (Mall and Galietta, 2015). Indeed, in human bronchial epithelial cells, it has been shown that SLC26A9 contributes to constitutive and cAMP-dependant chloride secretion (Bertrand et al., 2009; Avella et al., 2011). Physical interaction between SLC26A9 and CFTR has been shown in several studies (Bertrand et al., 2009; Chang et al., 2009; Avella et al., 2011), even if the consequences of this interaction [reviewed in (El Khouri and Toure, 2014)] are still controversial. According to these studies, SLC26A9 interaction with CFTR enhances CFTR activity (Bertrand et al., 2009; Avella et al., 2011). Reciprocally, CFTR has been shown to modulate SLC26A9 function (Bertrand et al., 2009). However, there has also been evidence showing that CFTR inhibits the activity of SLC26A9 (Chang et al., 2009; Ousingsawat et al., 2012). More recently, SLC26A9 membrane expression and activity was shown to be decreased by CFTR-F508del in co-expression experiments using HEK cells. Interestingly, the correction of F508del CFTR by VX-809 (lumacaftor) was also shown to restore SLC26A9 activity (Bertrand et al., 2017). Finally, a previous functional analysis of eight non-synonymous coding SNPs (p.Y70N, p.T1247N, p.I384T, p.R575W, p.606L, p.V622L, p.V744M, and p.H748R) revealed several functional modifications, including increased or decreased channel activity and altered protein expression that could modify disease (Chen et al., 2012).

**FIGURE 5:**
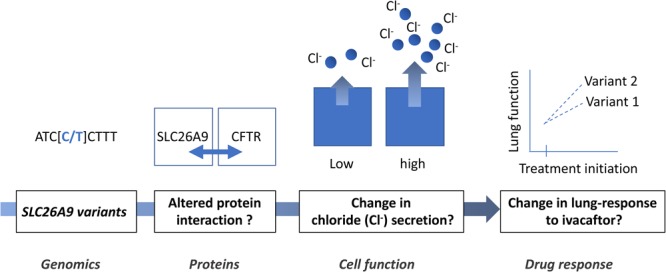
Proposed mechanism of the modulation of ivacaftor lung response in CF patients by *SLC26A9* gene.

The exact mechanism explaining how *SLC26A9* variants affect the ivacaftor responses that we observed in our patients is unknown (depicted in **Figure 5**) and requires future investigation. Based on previous reports, it is reasonable to hypothesize that the variants have an impact on SLC26A9-CFTR-G551D interactions, which could result in altered intracellular trafficking of CFTR-G551D and/or transporter activation.

Our study has several limitations, mainly related to the small sample size of the cohort treated with ivacaftor, due to the rarity of *CFTR* gating mutations (∼4% of individuals with CF). Moreover, baseline FEV_1_ measurements at ivacaftor treatment initiation was missing for about one third of the patients (see flowchart), which reduced even more the size of the analyzed cohort. Nevertheless, this study highlights that the interindividual variability in the lung response to ivacaftor might genetically be associated with *SLC26A9* variants in French CF patients and confirms the key pleiotropic role of this gene in CF. To confirm these results, it will be important to extend this study to patients with CF from different countries worldwide. The elucidation of the biological mechanisms beyond this variability is also necessary and will require functional genetic studies. In the exciting current era of curative treatment development in CF, pharmacogenomics will soon be an integral part of patient care, to modify treatment accordingly to provide the ultimate personalized medicine.

## Author Contributions

HC, P-YB, and LG designed the study and wrote the manuscript. P-YB, JM, and I-HD performed the data analysis. LS critically revised the manuscript. HC and JM participated in patient recruitment, sample collection, and phenotyping.

## Conflict of Interest Statement

The authors declare that the research was conducted in the absence of any commercial or financial relationships that could be construed as a potential conflict of interest.

## Footnotes

1 http://cftr2.org

2 www.ensembl.org

3 http://www.R-project.org/
