# Supplementary material for: SLC26A9 Gene Is Associated With Lung Function Response to Ivacaftor in Patients With Cystic Fibrosis
Source: Front Pharmacol. 2018 Jul 26;9:828. doi: 10.3389/fphar.2018.00828 (PMC6095007; doi:10.3389/fphar.2018.00828)
Supplement: Supplementary file 1 [file Table_1.DOC]

**Table S1. CFTR genotypes for the patients with cystic fibrosis** included in the ivacaftor lung response analysis

|  | | |
| --- | --- | --- |
| **Patients*** | ***CFTR* mutation 1** | ***CFTR* mutation 2** |
| 1 | G551D | 4016insT |
| 2 | F508del | G551D |
| 3 | F508del | G178R |
| 4 | F508del | G551D |
| 5 | F508del | G1244E |
| 6 | G551D | W1282X |
| 7 | S549N | Not available |
| 8 | G551D | 621+1G>A |
| 9 | W1282X | G1244E |
| 10 | F508del | G178R |
| 11 | F508del | G1244E |
| 12 | F508del | S1251N |
| 13 | F508del | S1251N |
| 14 | F508del | S1251N |
| 15 | G551D | Q220X |
| 16 | G551D | 574delA |
| 17 | F508del | G1244E |
| 18 | G551D | N1303K |
| 19 | F508del | G551D |
| 20 | F508del | G1244E |
| 21 | G551D | 4006-1G>A |
| 22 | S549R | 3121-1G>A |
| 23 | F508del | G551D |
| 24 | F508del | G551D |
| 25 | F508del | G551D |
| 26 | F508del | G178R |
| 27 | F508del | G551D |
| 28 | F508del | G551D |
| 29 | F508del | S1251N |
| 30 | F508del | G551D |

**CFTR* genotypes of the 30 patients with cystic fibrosis included in the ivacaftor lung response analysis according to the following inclusion criteria: pancreatic insufficient, carrying at least one ivacaftor-approved gating mutation (G551D, G1244E, G1349D, G178R, G551S, S1251N, S1255P, S549N, and S549R), baseline FEV_1pp_ between 40 and 90%, and available post-treatment FEV_1pp_ data.
