# Supplementary material for: SLC26A9 Gene Is Associated With Lung Function Response to Ivacaftor in Patients With Cystic Fibrosis
Source: Front Pharmacol. 2018 Jul 26;9:828. doi: 10.3389/fphar.2018.00828 (PMC6095007; doi:10.3389/fphar.2018.00828)
Supplement: Supplementary file 2 [file Table_2.docx]

**Table S2. Description of the *SLC26A9* variants**

| ***SLC26A9* variants** | **Position^£^** | **Allele** | | | |
| --- | --- | --- | --- | --- | --- |
|  |  | **m^*^** | **M^*^** | **MAF^**^** | **HWE^***^** |
| rs7512462 | (205899595) | C | T^(^°^)^ | 40.9 | 0.49 |
| rs1874361 | (205908186) | A | C^(^°^)^ | 45.4 | 0.83 |
| rs12741299 | (205914516) | T | C^(^°^)^ | 9.2 | 0.05 |
| rs4077468 | (205914757) | G^(^°^)^ | A | 40.8 | 0.98 |
| rs4077469 | (205914885) | T^(^°^)^ | C | 40.8 | 0.83 |
| rs12047830 | (205916699) | A | G^(^°^)^ | 48.6 | 0.93 |
| rs7419153 | (205917309) | A | G^(^°^)^ | 37.4 | 0.30 |

**^*^**m(inor), M(ajor), **^(^°^)^**Ancestral; ^**^Minor allele frequency (%); ^***^Hardy-Weinberg equilibrium P-value; **^£^**physical position according to ensembl GRCh37.
