# Supplementary material for: SLC26A9 Gene Is Associated With Lung Function Response to Ivacaftor in Patients With Cystic Fibrosis
Source: Front Pharmacol. 2018 Jul 26;9:828. doi: 10.3389/fphar.2018.00828 (PMC6095007; doi:10.3389/fphar.2018.00828)
Supplement: Supplementary file 3 [file Table_3.doc]

**Haplotypes’ analyses and results**

The 2 most common haplotypes reconstructed in our sample were the same as the phased haplotypes found in European participants to 1000 genomes (Table S3). Note that H1, the most common haplotype, comprised all SNPs alleles associated with a larger FEV_1pp_ change with treatment, while H2 comprised most alleles associated with reduced FEV_1pp_ change.

We analyzed FEV_1pp_ change according to haplotypes, coding the number of TACACGA haplotypes and the number of other haplotypes with respect to a reference individual carrying 2 CCCGTAG haplotypes. Overall, this analysis did not provide significant evidence for a heterogeneity in FEV_1pp_ change with haplotypes (P=0.2 over 15-75 days, P=0.08 over one year), but was underpowered given the large diversity of haplotypes in the sample. Yet, the direction of association for carrying one H2 instance, comprising SNP alleles with a reduced impact on FEV_1pp_ change, relative to H1, comprising SNP alleles with a positive impact on FEV_1pp_ change, agreed with the empirical changes reported in the manuscript: there was 10% less response (CI95%: [-1, +21]) with carriage of a TACACGA haplotype over 15-75 days (with respect to CCCGTAG) and likewise over one-year post treatment (13% less FEV_1pp_ change (CI95% [1, 25]).

**Table S3. Haplotypes distribution in patients carrying at least one ivacaftor-approved *CFTR* gating mutation in decreasing frequency (n=30).**

| **Haplotype** | **rs7512462** | **rs1874361** | **rs12741299** | **rs4077468** | **rs4077469** | **rs12047830** | **rs7419153** | **Percentage*** |
| --- | --- | --- | --- | --- | --- | --- | --- | --- |
| **H1** | T(+) | A(+) | C(+) | A(+) | C(+) | G(+) | A(+) | 35% |
| **H2** | C(-) | C(-) | C | G(-) | T(-) | A(-) | G(-) | 18% |
| **H3** | T | C | T(+) | A | C | G | A | 8% |
| **H4** | C | A | C | G | T | A | G | 7% |
| **H5** | T | A | C | A | C | A | G | 7% |
| **H6** | T | A | C | A | C | G | G | 7% |
| **H7** | C | C | C | A | C | A | G | 5% |
| **Other** |  |  |  |  |  |  |  | 13% |

*Haplotypes are listed if percentage > 5%. (+/-): direction of association with FEV_1pp_ change.
